# Mediating effect analysis: How frailty affects fear of falling and fall risk in elderly patients with ischemic stroke

**DOI:** 10.1097/MD.0000000000045035

**Published:** 2025-10-03

**Authors:** Mingjie Chen, Zhenhui Pan, Chuanhang Lin

**Affiliations:** a Zhaoqing First People’s Hospital, Zhaoqing City, Guangdong Province, China; b Hezhou People’s Hospital, Hezhou, Guangxi, China.

**Keywords:** elderly ischemic stroke, fall risk, fear of falling, frailty, mediating effect

## Abstract

This study aimed to examine how frailty affects fall risk in elderly ischemic stroke patients through the mediating role of fear of falling, by analyzing their associations and pathways. A total of 280 elderly ischemic stroke patients were recruited by convenience sampling. Evaluations included the Chinese versions of the elderly falls risk self-assessment scale, modified falls efficacy scale (MFES), tilburg frailty indicator (TFI), hospital anxiety and depression scale (HADS), and family APGAR index, all showing good reliability (Cronbach α ≥ 0.80). Data were analyzed using Pearson correlation, univariate analysis, and multiple linear regression. Age increased fall risk, while exercise and assistive devices reduced it (*P* < .0001). Multiple regression analysis confirmed the effects of age (β = 0.8233, *P* < .0001) and use of assistive devices (β = −0.0143, *P* < .0001). Scores across frailty dimensions (mean = 5.12) were strongly and positively associated with fall risk (*R* = 0.886, *P* < .001). Conversely, fear of falling showed a significant negative correlation with fall risk (*r* = −0.834, *P* < .001), suggesting that higher levels of fear may reduce actual fall events through defensive or avoidance behaviors. Frailty significantly increased fall risk in elderly patients with ischemic stroke, while fear of falling served as a partial mediator in this process. Greater frailty was associated with stronger fear of falling, which in turn contributed to an elevated fall risk. This underscores the importance of addressing both frailty and psychological factors (e.g., fear of falling) in clinical care for this population. Future interventions should target both physical frailty and fear of falling to reduce fall incidence, alleviate patients’ physical and psychological burdens, and improve safety and quality of life. The novelty of this study lies in being the first to construct and validate a frailty–fear of falling–fall risk mediation model in elderly patients with ischemic stroke, thereby providing new evidence on the mechanisms underlying fall risk in this group. The observed association may be partly explained by the fact that patients with higher fear levels tend to adopt more protective or avoidance behaviors.

## 1. Introduction

Ischemic stroke is a common chronic condition in the elderly, characterized by high rates of recurrence, disability, and mortality. Previous studies have shown that the recurrence rate within the first year can reach 20 to 30%,^[1]^ with approximately 50% of patients experiencing varying degrees of functional impairment.^[2]^ Mortality rates may reach 10 to 15% within the first month after onset and 18 to 44.6% within 90 days,^[1]^ seriously impairing patients’ quality of life and imposing significant economic and psychological burdens on patients and their families.^[1,2]^ Falls are one of the most frequent complications during the rehabilitation period of stroke patients. Studies have reported that 30 to 50% of stroke survivors experience falls within the first year after stroke onset, making falls an important factor hindering functional recovery. Furthermore, approximately 40 to 73% of stroke patients experience at least one fall within 6 months after discharge. Falls not only reduce independence but also further obstruct functional rehabilitation,^[3]^ while simultaneously increasing hospital readmission and mortality rates.^[4]^

In recent years, frailty has drawn increasing attention as a major determinant of fall risk in elderly patients with ischemic stroke.^[5,6]^ Previous evidence suggests that frail elderly patients are nearly twice as likely to experience falls after ischemic stroke compared to non-frail patients.^[7]^ Frailty is not only associated with physical decline but also encompasses changes in psychological and social dimensions.^[6]^ In addition, fear of falling plays a key role in shaping fall risk. Fear of falling refers to the persistent concern about falling during daily activities. Systematic reviews indicate that its prevalence among the elderly ranges from 20 to 85%, and specific studies show that 38 to 54% of older adults report continuous concern about falls in daily life.^[8]^ Such fear often results in avoidance behaviors, which reduce participation in physical and social activities. Cognitive assessments further reveal that greater fear of falling is associated with lower confidence and a diminished sense of control, which in turn negatively impacts behavior and activity levels.^[9,10]^ Existing studies suggest that frailty and fear of falling may interact through complex mechanisms to influence fall risk.^[11]^

However, the specific relationships between frailty and fall risk, and between fear of falling and fall risk in elderly patients with ischemic stroke, remain unclear. Therefore, this study aimed to examine the interrelationships among frailty, fear of falling, and fall risk in elderly patients with ischemic stroke using a cross-sectional design. The findings are expected to provide a theoretical basis for clarifying the mechanisms underlying falls in this population and offer guidance for fall prevention strategies. Compared with previous research, the innovations of this study are as follows: it specifically targeted elderly patients with ischemic stroke, focusing on this unique high-risk group; it employed a mediation model to systematically test the pathways linking frailty, fear of falling, and fall risk; and it incorporated social support, emotional factors, and medication use into the analysis, providing a more comprehensive evaluation of fall risk.

## 2. Methods

### 2.1. Study subjects

This study adopted a convenience sampling method to recruit 280 elderly patients with ischemic stroke who were hospitalized in the Department of Neurology of our hospital between January 2023 and June 2024.

Inclusion criteria: age ≥ 60 years; met the diagnostic criteria for ischemic stroke and confirmed by CT/MRI; clinically stable and able to cooperate with the investigation; complete clinical data; and patients provided informed consent and voluntarily participated in the study.

Exclusion criteria: patients with severe psychiatric disorders or those unable to cooperate with the investigation; patients who withdrew for personal reasons; and patients in the terminal stage of stroke or with severe critical conditions.

### 2.2. Research instruments

#### 2.2.1. General baseline questionnaire

Based on a comprehensive literature review and an in-depth understanding of the disease characteristics of elderly ischemic stroke patients, the researchers designed a general questionnaire tailored to clinical practice and research objectives. The questionnaire included 2 categories of information:

1. Sociodemographic characteristics: age, gender, education level, marital status, place of residence, living arrangement, primary caregiver, monthly household income, smoking history, alcohol consumption, physical activity level, sleep quality, and medical insurance payment method.
2. Clinical characteristics: comorbidities, body mass index, number of stroke episodes, duration of disease, use of assistive devices, activities of daily living, history of falls, regular medication use, types of medications, and stroke symptoms.

#### 2.2.2. Chinese version of the elderly falls risk self-assessment scale

This study employed the Chinese version of the elderly falls risk self-assessment scale adapted by Li and Ding.^[12]^ The scale, validated in inpatient, outpatient, and community elderly populations, demonstrated good reliability and validity. It contains 12 items, with items 1 and 2 scored from 0 to 2, and the remaining items scored from 0 to 1. The total score ranges from 0 to 14, with scores > 4 indicating a risk of falling. Higher scores correspond to higher fall risk. The Cronbach α coefficient was 0.92, showing excellent internal consistency.

#### 2.2.3. Chinese version of the modified falls efficacy scale (MFES)

The Chinese version of the MFES, adapted by Hao and Liu,^[13]^ was used in this study. The scale includes 14 items across 2 dimensions: 9 indoor items and 5 outdoor items. Each item is scored from 0 to 10, where 0 represents low efficacy and high fear of falling, 5 represents moderate efficacy and moderate fear, and 10 represents high efficacy and low fear. Higher total scores indicate stronger fall efficacy and lower fear of falling. The Cronbach α coefficient in this study was 0.93, indicating excellent internal consistency.

#### 2.2.4. Tilburg frailty indicator (TFI)

Frailty status was assessed using the tilburg frailty indicator (TFI) developed by Gobbens. This self-reported scale includes 3 dimensions: physical, psychological, and social frailty, with a total of 15 items. The total score ranges from 0 to 15, with scores > 5 indicating frailty, and higher scores representing greater severity. The Chinese version was translated and validated by Xi Xing^[14]^ in 2013 among elderly patients with chronic diseases, with a Cronbach α coefficient of 0.94, showing good reliability and validity.

#### 2.2.5. Hospital anxiety and depression scale (HADS)

The hospital anxiety and depression scale (HADS), developed by Snaith et al^[15]^ in 1983, was used to assess anxiety and depression. This widely applied scale includes 2 subscales: anxiety (HADS-A) and depression (HADS-D), each containing 7 items. Items 1, 3, 5, 7, 9, 11, and 13 assess depression, while items 2, 4, 6, 8, 10, 12, and 15 assess anxiety. Each item is scored from 0 to 3. A score of 0 to 7 indicates no symptoms, 8 to 10 suggests possible anxiety or depression, and 11 to 21 indicates definite anxiety or depression. Scores ≥ 8 are considered positive. In this study, the Cronbach α coefficient was 0.80, indicating good internal consistency.

#### 2.2.6. Family APGAR index

The family APGAR index, developed by Smilkstein,^[16]^ was applied to assess family function. It consists of 5 items rated on a 3-point Likert scale: “rarely” (0), “sometimes” (1), and “often” (2). The total score ranges from 0 to 10, with ≤ 3 indicating severe dysfunction, 4 to 6 indicating moderate dysfunction, and ≥ 7 indicating good family functioning. The Cronbach α coefficient was 0.83, demonstrating good internal consistency.

### 2.3. Sample size estimation

Sample size estimation was conducted using Kendall method,^[17]^ which recommends 5 to 10 times the number of independent variables for multivariate analysis. Allowing for the maximum ratio and 15% invalid questionnaires, the formula was:


$$\begin{array}{l} Sample\;size=number\;of\;independent\;variables\times \left(5\text{–}10\right)\times \left(1+15\%\right). \end{array}$$

With 28 independent variables, the estimated range was 161 to 322 participants. The final sample size was set at 280.

### 2.4. Data collection

This study was approved by the hospital ethics committee. Patients meeting inclusion and exclusion criteria were recruited, informed consent was obtained, and participants signed consent forms. Questionnaires were self-administered with on-site guidance from trained researchers, who avoided influencing responses to reduce bias. For patients unable to complete questionnaires due to physical disabilities, assistance was provided. Questionnaires were collected immediately after completion, and any missing data were clarified with patients on the spot to ensure completeness.

### 2.5. Quality control

1. Design phase: The questionnaires were constructed based on literature review and research objectives, selecting scales with proven reliability and validity. A pilot test was conducted prior to the formal survey, and adjustments were made according to feedback.
2. Implementation phase: Ethical approval and informed consent were obtained. All researchers received standardized training regarding the purpose, methods, and considerations of the survey to ensure consistency. Patients who were uncooperative or withdrew midway were recorded as dropouts.
3. Data analysis phase: Questionnaires were checked immediately upon collection for errors or omissions. Data entry was performed using double-checking to minimize errors. Suspicious data were reverified to ensure accuracy and completeness.

### 2.6. Statistical methods

Several statistical methods were employed to explore factors influencing fall risk in elderly ischemic stroke patients. Univariate analyses (*t*-test, ANOVA) were used to evaluate differences among groups. Multiple linear regression analysis was conducted to comprehensively assess the effects of multiple independent variables on fall risk, quantify the contribution of each factor, and test statistical significance. Cronbach α coefficient was used to evaluate the internal consistency of all scales, with values closer to 1 indicating higher reliability. A *P*-value <.05 was considered statistically significant.

## 3. Results

### 3.1. Factors influencing fall risk in elderly patients with ischemic stroke

#### 3.1.1. Univariate analysis of fall risk

In the univariate analysis of fall risk among 280 elderly patients with ischemic stroke, significant differences were observed across age groups, particularly between patients aged 60 to 70 years and those aged ≥ 81 years (*P* = .0019), indicating that fall risk increases with age. No significant associations were found between fall risk and variables such as gender, comorbidities, or body mass index (all *P* > .05).

Regarding physical activity, patients who exercised occasionally exhibited a significantly higher fall risk compared with those who exercised regularly or frequently (*P* = .00003), highlighting the protective effect of regular exercise. Activities of daily living were positively correlated with fall risk, with the fully dependent group showing the highest risk (*P* = .00001). A history of falls was also strongly associated with increased fall risk, with the fall-history group showing significantly higher risk than those without prior falls (*P* = .00001).

Furthermore, both the use of assistive devices and the types of medications were related to fall risk (*P* = .0014 and *P* = .035, respectively), suggesting that assistive device use and polypharmacy should be carefully considered in fall risk assessment. Anxiety and depression levels were closely associated with fall risk, with higher scores in these domains significantly increasing fall risk (*P* < .001). Patients with low social support also exhibited significantly higher fall risk compared with other groups (*P* = .00421).

Frailty status was another strong influencing factor, with severely frail patients showing markedly higher fall risk than those with mild or no frailty (*P* = .00001). These findings emphasize that multiple factors – including age, psychological status, and frailty level – play critical roles in determining fall risk among elderly patients with ischemic stroke (see Table 1).

**Table 1:** Univariate analysis of fall risk in elderly patients with ischemic stroke.

Variable	Grouping	N	Fall Risk (M ± SD)	*F*/*t*	*P*
Age	60–70	119	5.26 ± 2.42	6.44	.0019
	71–80	115	6.05 ± 2.75		
	≥81	46	6.88 ± 3.29		
Gender	Male	142	5.89 ± 2.79	0.38	.704
	Female	138	5.87 ± 2.82		
Comorbidity	0	9	6.31 ± 2.96	0.92	.4014
	1–2	241	5.81 ± 2.78		
	≥3	30	5.87 ± 2.84		
BMI	<18.5	9	3.81 ± 2.13	2.12	.1216
	18.5–23.9	139	6.01 ± 2.97		
	≥24	132	5.87 ± 2.82		
Education level	Junior high school or below	156	5.75 ± 2.53	2.32	.0755
	High school/Vocational school	69	5.61 ± 2.56		
	Associate degree	44	6.19 ± 3.95		
	Bachelor’s degree	11	6.08 ± 3.93		
Marital status	Married	211	5.73 ± 2.75	2.19	.1133
	Divorced/Separated	14	5.18 ± 3.07		
	Widowed	55	6.27 ± 3.03		
Residence location	Urban	160	5.82 ± 2.80	−1.19	.2339
	Rural	120	5.81 ± 2.80		
Living arrangement	Living alone	14	5.55 ± 3.37	1.34	.2640
	Living with spouse	176	5.83 ± 2.73		
	Living with children	90	5.82 ± 2.91		
Primary caregiver	Family member	254	5.88 ± 2.81	1.61	.2018
	Self	18	5.04 ± 2.54		
Monthly family income	Other	8	5.91 ± 3.01	2.15	.0943
	Below 2000 RMB	18	6.01 ± 3.10		
	2000–3999	129	6.29 ± 2.67		
	4000–5999	91	5.49 ± 2.76		
	Above 6000 RMB	42	5.30 ± 3.02		
Smoking history	Yes	48	5.56 ± 2.76	0.29	.7749
	No	232	5.86 ± 2.81		
Alcohol consumption history	Yes	31	5.57 ± 3.01	−0.06	.9519
	No	249	5.86 ± 2.79		
Level of physical activity	Occasionally (0–3 times/wk)	97	6.92 ± 2.47	13.26	.00003
	Often (4–6 times/wk)	99	5.62 ± 2.75		
	Frequently (≥7 times/wk)	84	4.90 ± 2.86		
Sleep condition	Easy to fall asleep	194	5.90 ± 2.80	0.99	.3734
	Difficulty falling asleep	31	5.39 ± 2.81		
	Vivid dreams and awakenings	55	5.80 ± 2.91		
Health insurance payment method	Urban and rural resident insurance	165	6.11 ± 2.76	0.69	.493
	Urban employee insurance	115	5.93 ± 2.84		
Number of disease episodes	1	151	5.76 ± 2.68	0.24	.787
	2–3	60	5.88 ± 3.01		
	≥3	69	5.91 ± 2.99		
Duration of stroke (mo)	<1 mo	145	5.68 ± 2.63	2.31	.058
	1 ≤ duration < 3	15	6.22 ± 3.41		
	3 ≤ duration < 6	9	5.90 ± 2.58		
	6 ≤ duration < 12	16	5.70 ± 2.94		
	≥12 mo	95	5.95 ± 2.99		
Use of assistive devices	Yes	115	6.51 ± 2.90	3.23	.0014
	No	165	5.34 ± 2.65		
Activities of daily living	Completely independent	75	4.56 ± 2.56	11.99	.00001
	Requires partial care	152	5.98 ± 2.83		
	Requires full care	53	7.08 ± 2.47		
History of falls	Yes	206	6.27 ± 2.74	4.44	.00001
	No	74	4.61 ± 2.67		
Regular medication use	Yes	221	5.92 ± 2.82	0.41	.682
	No	59	5.44 ± 2.72		
Types of medications	<3	132	5.40 ± 2.61	−2.12	.035
	≥3	148	6.15 ± 2.92		
Stroke symptoms(n)	0	13	6.07 ± 2.21	20.02	.0000
	1–3	232	5.53 ± 2.72		
	≥3	35	7.50 ± 2.86		
Level of family care	Good condition	148	5.96 ± 2.82	1.19	.307
	Moderate impairment	96	5.52 ± 2.76		
	Severe impairment	36	5.95 ± 2.81		
Anxiety	None	118	4.01 ± 2.42	86.90	.00000
	Mild	77	6.03 ± 2.21		
	Moderate	57	7.80 ± 1.63		
	Severe	28	9.16 ± 1.48		
Depression	None	116	.01 ± 2.34	51.95	.00000
	Mild	75	6.18 ± 2.04		
	Moderate	60	7.12 ± 2.50		
	Severe	29	9.17 ± 1.46		
Frailty	<5	101	3.11 ± 1.63	−18.15	.00000
	≥5	179	7.31 ± 2.10		
Fear of falling	<8	175	7.10 ± 2.17	13.55	.00000
	≥8	105	3.66 ± 2.40		

#### 3.1.2. Multivariate linear regression analysis of fall risk

A multivariate regression analysis was conducted to further examine the factors influencing fall risk in elderly ischemic stroke patients. Fall risk was used as the dependent variable, while independent variables included age, physical activity level, use of assistive devices, activities of daily living, history of falls, types of medications, stroke symptoms, anxiety, depression, frailty, and fear of falling. Variable coding was as follows: Age: 60 to 70 years (=1), 71 to 80 years (=2), ≥81 years (=3); Physical activity: occasional (0–3 times/week, =1), regular (4–6 times/week, =2), frequent (≥7 times/week, =3);Use of assistive devices: yes (=1), no (=2); Activities of daily living: fully independent (=1), partially dependent (=2), fully dependent (=3); Fall history: yes (=1), no (=2); Types of medications: <3 (=0), ≥3 (=1); Stroke symptoms: none (=0), 1–3 symptoms (=1), ≥4 symptoms (=2); Anxiety, depression, frailty, and fear of falling were entered as continuous measured values.

The results indicated that age had a significant positive effect on fall risk (β = 0.8233, *P* < .0001), confirming that fall risk increases with age. Use of assistive devices (β = −0.0143, *P* < .0001) significantly reduced fall risk, while a history of falls (β = 0.0027, *P* = .0350) significantly increased risk. Stroke symptoms (β = 0.0049, *P* < .0001) and depression (β = 0.0043, *P* < .0001) were also significantly associated with fall risk, suggesting that both disease severity and psychological health exert important effects.

Interestingly, anxiety was negatively correlated with fall risk (β = −0.0116, *P* < .0001), suggesting that patients with higher anxiety may adopt more precautionary behaviors, thereby reducing their actual risk of falling. Although the number of medications did not reach statistical significance (β = −0.0006, *P* = .682), its potential influence cannot be excluded, as suggested by the univariate findings. Detailed results are presented in Table 2.

**Table 2:** Multivariate linear regression analysis of fall risk in elderly patients with ischemic stroke.

Variable	(β)	SE	*t*	*P*	95% Confidence interval
Constant	4.5133	0.0070	615.9150	.0000	[4.4996, 4.5270]
Age	0.8233	0.0010	678.6040	.0000	[0.8213, 0.8253]
Level of Physical Activity	−0.0019	0.0020	−1.1200	.2640	[−0.0058, 0.0020]
Use of assistive devices	−0.0143	0.0020	−7.9770	.0000	[−0.0182, −0.0104]
Activities of daily living	0.0021	0.0010	2.7690	.0060	[0.0001, 0.0041]
History of falls	0.0027	0.0010	2.1170	.0350	[0.0007, 0.0047]
Types of medications	−0.0006	0.0020	−0.4100	.6820	[−0.0045, 0.0033]
Stroke symptoms	0.0049	0.0010	4.6650	.0000	[0.0029, 0.0069]
Anxiety	−0.0116	0.0010	−9.3840	.0000	[−0.0136, −0.0096]
Depression	0.0043	0.0010	5.0390	.0000	[0.0023, 0.0063]
Frailty	−0.0013	0.0000	−3.0140	.0030	[−0.0013, −0.0013]
Fear of falling	−0.0053	0.0010	−7.2480	.0000	[−0.0073, −0.0033]

### 3.2. Relationship between frailty, fear of falling, and fall risk in elderly patients with ischemic stroke (cross-sectional study)

In the assessment of frailty, fear of falling, and fall risk among elderly patients with ischemic stroke, the results showed the following mean scores across different dimensions of frailty: the physical dimension scored 5.12 (±2.08), the psychological dimension 2.06 (±1.03), and the social dimension 1.26 (±0.65), indicating that physical frailty was generally more pronounced, whereas psychological and social frailty were relatively lower. The mean score for fear of falling in the indoor dimension was 6.55 (±1.97), while the outdoor dimension averaged 5.45 (±2.36), suggesting that patients’ fear of falling varied across different environments. The average fall risk score was 5.83 (±2.83), reflecting that elderly patients generally faced a notable level of fall risk (see Table 3).

**Table 3:** Scores for frailty, fall fear, and fall risk in elderly patients with ischemic stroke.

Variable	Dimension	Item count	Minimum value	Maximum value	Score range	Average score
Frailty		15	0	10	0–15	5.12 ± 2.08
	Physical	8	0	6	0–8	2.06 ± 1.03
	Psychological	4	0	3	0–4	1.82 ± 0.85
	Social	3	0	3	0–3	1.26 ± 0.65
Fear of falling		14	32	140	0–140	6.55 ± 1.97
	Indoor dimension	9	20	90	0–90	7.16 ± 1.89
	Outdoor dimension	5	8	50	0–50	5.45 ± 2.36
Fall risk		12	0	10	0–14	5.83 ± 2.83

Pearson correlation analysis revealed a strong positive correlation between fall risk and frailty, with a correlation coefficient of 0.886 (*P* < .001). Specifically, fall risk was moderately correlated with physical frailty (*R* = 0.727, *P* < .001), psychological frailty (*R* = 0.648, *P* < .001), and social frailty (*R* = 0.610, *P* < .001), all of which were statistically significant (see Table 4).

**Table 4:** Pearson correlation analysis of frailty and fall risk in elderly patients with ischemic stroke.

Variable	Fall risk	Frailty	Physical	Psychological	Social
Fall risk	1	0.886	0.727	0.648	0.610
Frailty	0.886	1	0.623	0.562	0.531
Physical	0.727	0.623	1	0.531	0.468
Psychological	0.648	0.562	0.531	1	0.382
Social	0.610	0.531	0.468	0.382	1

In the Pearson correlation analysis of fear of falling and fall risk, a strong negative correlation was observed (*r* = −0.834, *P* < .001), indicating that higher levels of fear of falling were associated with relatively lower fall risk. Fall risk was positively correlated with indoor activity levels (*R* = 0.814, *P* < .001) and outdoor activity levels (*R* = 0.799, *P* < .001), suggesting that increased activity may be associated with higher fall risk. Fear of falling was strongly negatively correlated with both indoor and outdoor activity levels (*r* = −0.975 and −0.960, respectively; both *P* < .001), indicating that greater fear of falling significantly reduced activity participation. Moreover, indoor and outdoor activity levels were highly positively correlated (*R* = 0.937, *P* < .001), demonstrating consistency in activity patterns across environments. These findings highlight the critical role of fear of falling and activity participation in influencing fall risk among elderly patients (see Table 5).

**Table 5:** Pearson correlation analysis of fall fear and fall risk in elderly patients with ischemic stroke.

Variable	Fall risk	Fear of falling	Indoor dimension	Outdoor dimension
Fall risk	1	−0.834	0.814	0.799
Fear of falling	−0.834	1	−0.975	−0.96
Indoor dimension	0.814	−0.975	1	0.937
Outdoor dimension	0.799	−0.96	0.937	1

Fall risk and fear of falling: The correlation was −0.834, indicating a strong negative relationship, suggesting that as fear of falling increases, fall risk relatively decreases. Fall risk and indoor dimension: The correlation was 0.814, indicating a strong positive relationship, meaning that higher levels of indoor activity may be associated with an increased fall risk. Fall risk and outdoor dimension: The correlation was 0.799, slightly lower than the indoor dimension, showing a moderate positive relationship with fall risk. Fear of falling and indoor/outdoor dimensions: The correlations were −0.975 and −0.960, respectively, both indicating strong negative relationships, suggesting that greater fear of falling may reduce both indoor and outdoor activity levels. Indoor and outdoor dimensions: The correlation was 0.937, showing a strong positive relationship, indicating that the trends of indoor and outdoor activity levels are generally consistent.

In the correlation analysis between frailty and fear of falling, significant associations were also identified. The strongest correlation was between physical condition and frailty (*R* = 0.864, *P* < .001), followed by psychological factors (*R* = 0.744, *P* < .001) and social factors (*R* = 0.701, *P* < .001), indicating that physical, psychological, and social conditions all contribute to frailty. Frailty was negatively correlated with indoor activity levels (*r* = −0.591, *P* < .001) and outdoor activity levels (*r* = −0.624, *P* < .001), suggesting that greater frailty was associated with reduced activity. Finally, frailty was strongly negatively correlated with fear of falling (*r* = −0.700, *P* < .001), the strongest negative relationship among all dimensions, further underscoring the close association between frailty and fear of falling. These results emphasize the importance of frailty, psychological status, and social factors in shaping fall risk among elderly ischemic stroke patients (see Table 6).

**Table 6:** Pearson correlation analysis of frailty and fall fear in elderly patients with ischemic stroke.

Variable	Frailty	Physical	Social	Psychological	Indoor dimension	Outdoor dimension	Fear of falling
Frailty	1	0.864	0.701	0.744	−0.591	−0.624	−0.700
Physical	0.864	1	0.684	0.671	−0.515	−0.516	−0.611
Social	0.701	0.684	1	0.594	−0.374	−0.417	−0.446
Psychological	0.744	0.671	0.594	1	−0.452	−0.412	−0.459
Indoor dimension	−0.591	−0.515	−0.374	−0.452	1	0.376	0.375
Outdoor dimension	−0.624	−0.516	−0.417	−0.412	0.376	1	0.477
Fear of falling	−0.700	−0.611	−0.446	−0.459	0.375	0.477	1

Frailty and other dimensions: Physical: Highest correlation, *R* = 0.864, *P* < .001, indicating a strong association between physical status and frailty. Social: *R* = 0.701, *P* < .001, showing that social factors have a significant influence on frailty. Psychological: *R* = 0.744, *P* < .001, indicating a significant positive correlation between psychological factors and frailty. Indoor dimension: Negative correlation, *r* = −0.591, *P* < .001, suggesting that increased frailty may be accompanied by reduced indoor activity. Outdoor dimension: Negative correlation, *r* = −0.624, *P* < .001, indicating a significant negative relationship between frailty and outdoor activity. Fear of falling: Negative correlation, *r* = −0.700, *P* < .001, representing the strongest negative association with frailty, indicating a close relationship between increased frailty and fear of falling.

### 3.3. Mediation effect of fear of falling between frailty and fall risk

**Figure 1.**
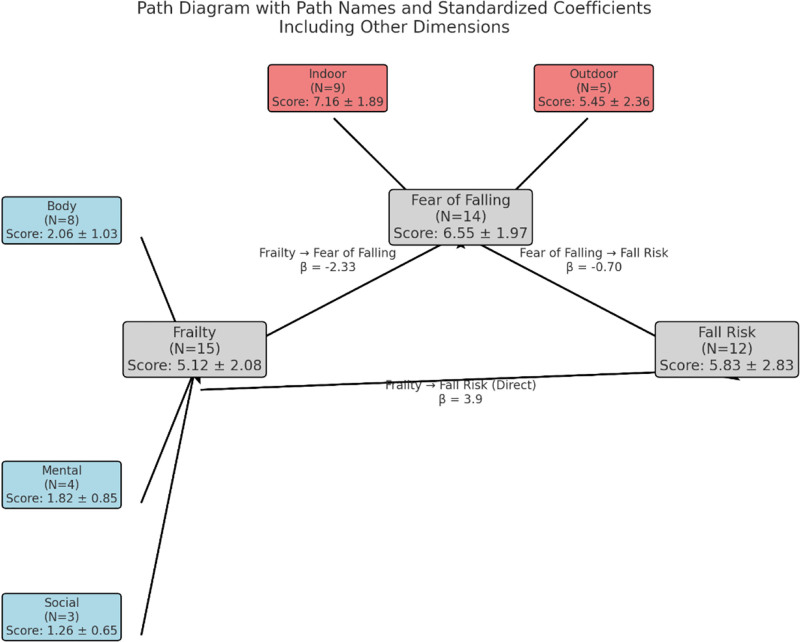
Mediating effect model diagram.

To clarify the pathway relationships among frailty, fear of falling, and fall risk in elderly ischemic stroke patients, we hypothesized that frailty directly influences fall risk, while also indirectly affecting fall risk through fear of falling, thereby suggesting a mediating role of fear of falling. Data analysis was performed using Python, with frailty and its 3 dimensions (physical, psychological, and social) as independent variables, fall risk as the dependent variable, and fear of falling and its 2 dimensions (indoor and outdoor) as mediating variables. The theoretical mediation model is shown in Figure 1.

The mediation model demonstrated good overall model fit (see Table 7).

**Table 7:** Model fit indices.

Fit index	X^2^/df	RMSEA	GFI	AGFI	NFI	CFI	IFI
Standard	<3	<0.08	>0.9	>0.8	>0.7	>0.7	>0.9
Model	1.635	0.055	0.987	0.956	0.987	0.987	0.987

The results indicated that frailty significantly predicted fear of falling (coefficient = −2.33), fear of falling significantly predicted fall risk (coefficient = −0.70), and frailty directly exerted a significant positive effect on fall risk (coefficient = 3.9). Path coefficients are detailed in Table 8.

**Table 8:** Path analysis of the mediating effect model.

Path	β	SE	CR	*P*
Frailty -> Fear of falling	−2.33	0.30	−7.77	<.001
Fear of falling -> Fall risk	−0.70	0.01	−2.80	.006
Frailty -> Fall risk (direct)	3.9	0.05	5.73	<.001

The mediating effect of fear of falling in the relationship between frailty and fall risk had an effect size of 0.372, accounting for 26.44% of the total effect. The 95% confidence interval (0.154, 0.595) did not include zero, indicating statistical significance. This suggests that fear of falling plays a partial mediating role between frailty and fall risk in elderly ischemic stroke patients (see Table 9).

**Table 9:** Mediating effect analysis of fall fear.

Path	Effect value	SE	95% Confidence interval	*P*	Effect proportion
Indirect effect of fear of falling	0.372	0.0139	(0.154, 0.595)	.000	26.44%
Direct effect	1.035	0.0815	(0.925, 1.145)	.000	73.56%
Total effect	1.407	0.0826	(1.304, 1.512)	.000	

## 4. Discussion

In this study, the frailty score among elderly ischemic stroke patients was 5.12 ± 2.08, with a frailty prevalence of 63.9%. This result is consistent with a meta-analysis^[6]^ reporting frailty prevalence in ischemic stroke patients ranging from 11.6 to 68.4%. These findings highlight that frailty is a serious issue in this population, underscoring the urgent need for appropriate interventions and management strategies. Specifically, the scores of different frailty dimensions followed an ascending order from social, psychological, to physical frailty. Among the elderly, the decline in physical function is often accompanied by reduced social support and deteriorating psychological health, which interact to form the complex mechanism of frailty,^[18]^ alongside the increasing number of chronic disease complications. Stroke-related impairments – such as motor dysfunction, speech and swallowing difficulties, and impaired balance – severely compromise physiological reserve and daily independence, thereby aggravating physical frailty.^[19]^ Physical impairments not only affect the physiological level but also exacerbate psychological burden, leading to negative emotions such as anxiety, depression, and fear of falling. These psychological problems further impact patients’ self-perception, resulting in excessive activity restriction and abnormal gait, which in turn intensify psychological frailty.^[20,21]^ Therefore, clinicians should closely monitor frailty status in elderly ischemic stroke patients, conduct early assessments, and develop individualized interventions to improve their overall quality of life.

Regarding fear of falling, this study found a mean score of 6.55 ± 1.97, which, although below the clinical cutoff value of 8, still reflects a relatively high level. This may be closely related to stroke-induced activity limitations and motor dysfunction. Previous studies have shown a close association between gait abnormalities and fear of falling, with fear potentially influencing neural control of gait, thereby exacerbating gait instability and fall risk.^[22]^ In this study, 62.5% of patients exhibited varying degrees of fear of falling. Further analysis revealed that the mean score for outdoor activity (5.45 ± 2.36) was lower than for indoor activity (7.16 ± 1.89), suggesting that patients had greater fear in outdoor environments. This may be attributed to the complexity of outdoor settings, which increases psychological stress and decreases activity confidence.^[23,24]^ By contrast, indoor environments offer a greater sense of security, which helps to alleviate negative emotions such as tension and anxiety, thereby reducing fear of falling.^[25]^ Fall risk in the cohort averaged 5.83 ± 2.83, reflecting that ischemic stroke patients are generally at high risk due to motor, sensory, and cognitive impairments that reduce balance and mobility.^[26,27]^ Prior studies reported that within 6 months after stroke, 12.5% of patients experienced a single fall and 13.5% had recurrent falls, while 15.9% of patients in inpatient rehabilitation sustained falls during hospitalization.^[26,27]^ The present study demonstrated a significant positive correlation between frailty and fall risk, with the strongest association in the physical domain, followed by psychological and social dimensions. This suggests that deterioration of physical function and negative psychological states are critical determinants of fall risk in elderly stroke patients.^[28]^

Pearson correlation analysis revealed a significant negative association between fear of falling and fall risk, indicating that patients with higher levels of fear may adopt more protective or avoidance behaviors, thereby lowering their actual fall risk. This finding aligns with Liu research.^[29]^ Previous studies also reported that fear of falling contributes to anxiety, depression, and reduced confidence in activity participation,^[30,31]^ negatively affecting rehabilitation and quality of life. The findings further indicated a strong association between frailty and fear of falling. Higher levels of physical frailty were linked to more severe fear of falling, consistent with previous studies.^[32,33]^ Strong correlations were found between fear of falling and both the physical and psychological domains of frailty. This may be explained by the common co-occurrence of symptoms such as dizziness, motor dysfunction, and visuospatial deficits in elderly ischemic stroke patients, which lead to reduced muscle strength and balance, thereby increasing fall risk and intensifying fear of falling.^[34,35]^ In addition, frailty may prolong disease recovery, diminish physiological reserves, reduce coping ability, and predispose patients to anxiety and depression.^[36,37]^ Therefore, clinical care should focus on assessing both frailty and fear of falling, encouraging active communication with patients and families to build confidence, and designing individualized rehabilitation programs with appropriate functional exercises.

This study showed that fear of falling partially mediated the relationship between frailty and fall risk, with a mediation effect of 0.372 (*P* < .0001). This indicates that frailty not only directly predicts fall risk but also indirectly influences it through fear of falling. Falls have a profound impact on prognosis, increasing hospital readmission rates and mortality. Therefore, monitoring and preventing fall risk must be a high clinical priority.^[38]^ The mediation model demonstrated that frailty exerts a direct predictive effect on fall risk, as elderly ischemic stroke patients, with reduced physiological reserves, are more vulnerable to stressors, leading to increased fall risk, readmission, and mortality.^[38,39]^

Frailty is reversible, preventable, and modifiable. Early management of frailty can reduce complications, lower recurrence rates, shorten hospital stays, and promote functional recovery, thereby improving prognosis. In this study, 26.44% of the effect of frailty on fall risk was mediated by fear of falling, underscoring its indirect predictive role. Elderly stroke patients with frailty often suffer from impaired balance, reduced muscle strength, and decreased attention, which aggravate psychological burden, reduce exercise confidence, and increase fear of falling.^[10]^

In addition, this study found an association between the number of medications and fall risk, suggesting that polypharmacy may be an important risk factor for falls in elderly ischemic stroke patients. Previous studies reported that polypharmacy not only directly increases fall risk but also exacerbates fear of falling through side effects such as dizziness and hypotension (BMC Geriatrics, 2024, doi:10.1186/s12877-024-04882-w).^[33]^ However, as this study did not perform detailed stratified analyses of specific drug types and comorbidities, further studies are warranted to clarify the underlying mechanisms.

Based on the mediation analysis, several clinical implications can be drawn. First, healthcare providers should prioritize early frailty assessment and management, tailoring rehabilitation plans and encouraging moderate physical activity to enhance both physical fitness and psychological confidence. Second, health education and psychological counseling should be employed to reduce patients’ psychological burden and promote positive attitudes. Third, multidisciplinary collaboration is essential to provide comprehensive care. On this basis, beyond conventional rehabilitation and psychological interventions, innovative technologies are increasingly being explored for fall risk management. Wearable sensors and intelligent monitoring systems can capture real-time gait, balance, and activity patterns to identify high-risk individuals.^[40]^ In addition, clinical trials integrating telemedicine with smart interventions have shown promising outcomes.^[41]^ Future research should investigate how these technologies can be combined with frailty and psychological assessments to achieve more precise fall risk prediction and individualized interventions for elderly ischemic stroke patients.

Finally, several limitations must be acknowledged. First, the sample size may limit generalizability and statistical power. Second, the cross-sectional design precludes causal inference, restricting findings to correlation only. Third, reliance on self-reported data for frailty and fear of falling may introduce reporting bias. Fourth, the lack of longitudinal follow-up prevents evaluation of the sustainability of interventions. Fifth, regional limitations may affect external validity. Finally, the absence of detailed analyses of comorbidities and specific drug categories may underestimate their role in the frailty–fall risk relationship. Future studies should adopt larger samples and incorporate polypharmacy, comorbidity data, and emerging technologies into multidimensional predictive models to improve the accuracy of fall risk assessment.

## 5. Conclusion

This study demonstrated that frailty and fear of falling significantly increase fall risk in elderly ischemic stroke patients. Greater frailty was associated with a higher likelihood of falls. Therefore, clinical care should emphasize comprehensive assessment and intervention for this population, with particular focus on frailty and psychological aspects. Strengthening physical exercise, providing psychological support, and enhancing family involvement are recommended to improve patients’ confidence and independence, thereby reducing fall risk. The innovation of this study lies in the first construction and validation of a mediation model of “frailty–fear of falling–fall risk” among elderly ischemic stroke patients, providing new evidence for the mechanisms underlying post-stroke fall risk and informing individualized intervention strategies.

## Author contributions

**Writing – original draft:** Mingjie Chen.

**Writing – review & editing:** Zhenhui Pan, Chuanhang Lin.
